# The Principles of Medical Ethics

**Published:** 1903-09

**Authors:** 


A Monthly Review of Medicine and Surgery.

*EDITOR:*

WILLIAM WARREN POTTER, M. D.

All communications, whether of a literary or business nature, books for review and exchanges should be addressed to the editor: 284 FRANKLIN STREET, BUFFALO, N. Y.

VOL. XLIII.—LIX. SEPTEMBER, 1903. No. 2.

### The Principles of Medical Ethics.

MISUNDERSTANDING and misrepresentation are two evils that are hard to eradicate. The second is apt to follow the first, though sometimes misrepresentation takes a tour on its own hook, and when it does it is not easy to overtake. Of late we have published in these columns considerable material bearing on the questions involved in the title at the head of this article, our object having been to try and throw some light on the somewhat anomalous conditions that exist amongst the medical profession in this state, relating to each other and to the general profession at large, represented in the state and national medical associations.

Nowhere, however, have we seen a better exposition of this subject than in the following editorial and letter, which we take great pleasure in reproducing from the *Medical Record* of August 8, 1903. We ask for it a patient reading by all who have any interest whatever in medical unity:

THE PRINCIPLES OF MEDICAL ETHICS IN THE UNITED STATES.

There has been much misunderstanding concerning the attitude of the Medical Society of the State of New York on the Code of Ethics question, for the original arguments upon which the differences of opinion regarding the desirability of modifying some of the provisions of the old rules have become now a part of ancient history. For this reason we take the opportunity of printing the following letter from our distinguished townsman, Dr. D. B. St. John Roosa, contributed to a recent issue of *The Lancet,* giving an account of the fundamental objects that actuated the movement. We do this the more willingly as it presents the views of the *Medical Record* on the subject, advocated at the time when the discussion was at its height and when party feeling resulted in the formation of radically opposed factions. Happily the recent action of the American Medical Association has reconciled all previous disagreements and has fallen in line with the advanced views regarding the relations of legally qualified practitioners to each other. Thus recent events have proved that the Medical Society of the State of New York was right in its original position, although much in advance of professional opinion at the time.

The following is the letter of Dr. Roosa:

"While making a short stay in London, in reading *The Lancet* of July 4, my attention was arrested by the views of your New York correspondent on the subject of the agitation concerning the code of ethics of our profession in the United States which has been more or less existent since 1882. Quite an opposite view from that taken by your correspondent is entertained by a large number of the profession, especially in the State of New York, on some points of his discussion. I therefore beg, as one of the active participants in this agitation, that I may be allowed briefly to present that view.

"The Medical Society of the State of New York, a representative and incorporated body of the regular medical profession which has been in existence for nearly 100 years, in the year 1882, having hitherto adopted the code of ethics of the American Medical Association, changed this code, chiefly in the matter of freedom in consultation with all legalised practitioners. The state society was led to this action by the fact that the state, in consequence of the expulsion of homeopathic practitioners from the regular county medical societies of the state some years before, had legalised these practitioners and organised them into a body with powers equal to those of the state society organised early in the last century. Many in the state society at that time advocated the abolition of all *written* codes of medical ethics. In the face of great opposition in other states and some in our own, the New York State Society in 1886 went further and abolished all reference to a code in its by-laws. But that society and all the societies represented in it, the New York Academy of Medicine being one of them, has ever since, in spite of the absence of a written code, maintained the highest standard of professional ethics.

"The object of this important action of the state society was not to secure consultations with homeopathic practitioners, but to establish a *modus vivendi* with them preliminary to securing their coöperation in the passage of a law making the same requirements of all practitioners of the state as to qualifications for practice within the state. The result was accomplished. Having been placed on an equal footing with the regular profession by that profession itself, as they had been already by the state, the Homeopathic State Society united with that of the regulars in securing one of the best laws that exists in any country regulating the licensing of practitioners of medicine. This law came into force in 1891. Now, no men or women can practise medicine in our state, no matter what degree or degrees they may have, unless they have passed a satisfactory and rigorous examination on all points in medical science.

"This examination is conducted by an independent board of medical examiners appointed by the Board of Regents of the University of the State. Homeopaths, regular practitioners, eclectics, even members of the colleges of physicians and of surgeons in London, graduates of Paris or Berlin, physicians from other states of the union, all must pass this state examination or not be allowed to practise in our borders. The only exception made as to uniformity in the examinations is that homeopathic and eclectic practitioners may be examined with different questions in therapeutics. In other respects the papers are of the same character. This law has been copied in other states of our country, notably by Pennsylvania, Virginia, Illinois, and others. It will be seen by this statement of facts that the Medical Society of the State of New York had a much broader aim than that of 'mitigating the restrictions of the old code' in abolishing all written codes of etiquette, and that its action has been followed by the most beneficent results to the profession, results which were fully in the minds of its members when they took upon themselves the responsibility and breasted the obloquy which their action involved. That action has finally been fully vindicated by the sentiments of the various states of the union as represented in the American Medical Association. This association has hitherto refused representation to the Medical Society of the State of New York, but at the next meeting that representation will undoubtedly be accorded and the strife over the code be ended."

